# Broadband high-efficiency near-infrared graphene phase modulators enabled by metal–nanoribbon integrated hybrid plasmonic waveguides

**DOI:** 10.1515/nanoph-2021-0709

**Published:** 2021-12-21

**Authors:** Longfang Ye, Kouxiang Yuan, Chunhui Zhu, Yao Zhang, Yong Zhang, Kunzhong Lai

**Affiliations:** Institute of Electromagnetics and Acoustics, Xiamen University, Xiamen 361005, China; Shenzhen Research Institute of Xiamen University, Shenzhen 518057, China; School of Electronic Science and Engineering, University of Electronic Science and Technology of China, Chengdu 611731, China

**Keywords:** graphene, integrated optics devices, modulators, waveguides

## Abstract

The phase modulator is a key component in optical communications for its phase modulation functions. In this paper, we numerically demonstrate a variety of ultra-compact high-efficiency graphene phase modulators (GPMs) based on metal–nanoribbon integrated hybrid plasmonic waveguides in the near-infrared region. Benefiting from the good in-plane mode polarization matching and strong hybrid surface plasmon polariton and graphene interaction, the 20 μm-length GPM can achieve excellent phase modulation performance with a good phase and amplitude decoupling effect, a low insertion loss around 0.3 dB/μm, a high modulation efficiency with V_*π*_L_*π*_ of 118.67 V μm at 1.55 μm, which is 1–3 orders improvement compared to the state-of-the-art graphene modulators. Furthermore, it has a wide modulation bandwidth of 67.96 GHz, a low energy consumption of 157.49 fJ/bit, and a wide operating wavelength ranging from 1.3 to 1.8 μm. By reducing the overlap width of the graphene–Al_2_O_3_–graphene capacitor, the modulation bandwidth and energy consumption of the modulator can be further improved to 370.36 GHz and 30.22 fJ/bit, respectively. These compact and energy-efficient GPMs may hold a key to various high-speed telecommunications, interconnects, and other graphene-based integrated photonics applications.

## 1 Introduction

As one of the crucial components for optical communications [1, 2], sensing [3], and integrated optical interconnections and circuits [4], optical modulators have gained considerable attention in recent years [5]. Taking the advantage of the amplitude or phase modulation schemes of high-efficiency optical modulators, the spectral efficiency and transmission rates for the communication systems can be greatly enhanced. One mainstream route to achieving efficient high-speed modulators is based on silicon photonics (SiPh) using the plasma dispersion effects, which has been commercially implemented in various communication links [6]. However, these all-silicon modulators still suffer from some drawbacks like limited modulation efficiency, optical bandwidth, large footprint, and inherent amplitude and phase modulation coupling [6]. To further enhance the modulation performance, many materials with strong electro-optic (electro-refractive, or electro-absorptive) effects such as germanium, ferroelectrics, III–V semiconductors, organic electro-optic materials, and 2D materials, have been investigated and integrated into the SiPh platform. Especially, the integration of new 2D materials may provide promising routes for high-performance modulators to meet the blistering surge in transmission capacity for optical interconnections and communications.

Graphene, an anatomically thick two-dimensional carbon nanomaterial, has attracted considerable interest because of its excellent electrical and optical properties, high carrier mobility, good thermal conductivity, large optical modulation, high-speed operation, and good SiPh compatibility [7], [8], [9]. This makes it an ideal electro-optic material for efficient amplitude and phase modulators. In recent years, a lot of graphene amplitude modulators have been investigated and developed. For example, Liu et al. first experimentally demonstrate graphene optical amplitude modulators with a modulation depth of ∼0.1–0.16 dB/μm [10, 11]. Since then, many different modulators based on single/double-layer graphene integrated with high refractive index waveguide core, metal ribbons, or photonic crystal structures have been proposed to improve the light confinement, enhance light-graphene interaction, and increase modulation depth while maintaining acceptable insertion loss [12], [13], [14], [15], [16], [17], [18]. On the other hand, graphene has also been introduced and integrated into various waveguide structures to achieve high-efficient phase modulators, which are crucial for encoding the phase information in complex formats [5, 13, 19], [20], [21], [22]. For example, in 2015, Sorianello et al. numerically demonstrated two phase modulators based on graphene–insulator–silicon capacitor and graphene–insulator–graphene capacitor with a modulation efficiency, namely the production of π-phase-shift voltage and length (V_*π*_L_*π*_), of 1600 V μm and 1000 V μm at 1.55 μm, respectively [13]. Then, in 2017, they experimentally demonstrated a graphene-silicon phase modulator with V_*π*_L_*π*_ of 2800 V μm [5]. In 2018, Shu et al. proposed a compact graphene modulator based on silicon waveguide using the electro refractive effect of graphene with V_*π*_L_*π*_ of 1290 V μm [19]. Moreover, Mao et al. proposed a low voltage and ultrafast graphene integrated phase modulator on semiconductor and dielectric platforms with V_*π*_L_*π*_ of 2150 V μm [20]. Despite the recent progress, the performance of most of the reported graphene phase modulators (GPMs) is still limited. Specifically, the modulation efficiency is restricted because of the large footprint and the weak light-graphene interaction caused by the mode polarization mismatch between them. It is remarkable that a longer phase modulator length allows a larger phase shift but inevitably induces higher insertion loss. There is a trade-off between optical loss, footprint, and V_*π*_L_*π*_ for the current GPMs. Therefore, how to drastically increase the light-graphene interaction and develop broadband high-performance GPMs with much higher modulation efficiency, smaller modulation length, and lower energy consumption, as well as maintain excellent decoupling between phase and amplitude modulation in the near-infrared region remains a challenge.

In this paper, we demonstrate a new type of high-efficiency GPM based on a dual-semicircular-metal–nanoribbon integrated graphene–insulator–graphene capacitor hybrid plasmonic waveguide structure for near-infrared applications. In this design, the surface plasmon polariton mode confinement, polarization matching (in-plane electric field components of graphene), and light-graphene interactions are drastically enhanced, enabling excellent phase modulation performance. To investigate the proposed modulator, we study and discuss the electric field distributions, the effective index Re(*n*_eff_), attenuation constant, phase change, the geometrical parameter effects, and the 3 dB modulation bandwidth characteristics at 1.55 μm. After dimensional optimization, the short 20 μm GPM shows a low optical loss around 0.3 dB/μm, a large 3 dB modulation bandwidth of 67.9 GHz, a low energy consumption of 157.49 fJ/bit, and a wide optical wavelength ranging from 1.3 to 1.8 μm. The GPM demonstrates a good phase and amplitude decoupling effect and a very high modulation efficiency with a small V_*π*_L_*π*_ of 118.6 V μm by changing the graphene chemical potential from 0.55 to 0.75 eV at 1.55 μm, which can be implemented in various efficient Mach–Zehnder (MZ) modulator. Especially, by reducing the overlap width of the graphene–Al_2_O_3_–graphene capacitor, the modulation bandwidth and energy consumption of the modulator can be further improved to 370.36 GHz and 30.22 fJ/bit, respectively. Finally, we present a comparison of the performance of the recently reported phase modulators. This work provides a new path for the design of high-efficiency GPMs relied on hybrid plasmonic effects and may have great potentials in near-infrared interconnects and telecommunication applications.

## 2 Design and consideration

**Figure 1:**
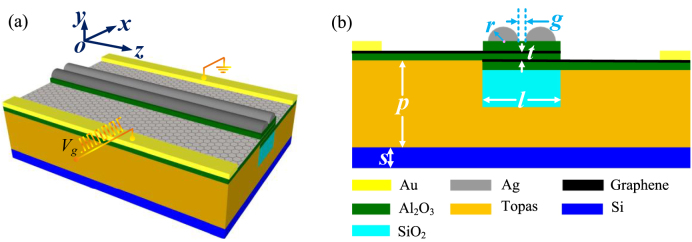
Schematic configuration of the GPM. (a) 3-D schematic illustration, (b) cross-sectional view. The structure dimensions are set as *s* = 100 nm, *p* = 800 nm, *l* = 600 nm, *t* = 5 nm, *r* = 100 nm, and *g* = 10 nm.

The 3D schematic configuration and 2D cross-sectional view of the proposed GPM are shown in Figure 1a and b, respectively. In this design, the modulator structure is made up of a dual-semicircular-silver-nanoribbon integrated graphene–insulator (Al_2_O_3_)–graphene capacitor placed above a SiO_2_ waveguide on a Topas buffer layer and a silicon substrate. The silicon substrate layer is with a thickness of 100 nm and a relative permittivity $\left(\epsilon_{si}\right)$ of 11.7. The Topas buffer layer is with a relative permittivity $\left(\epsilon_{\text{Topas}}\right)$ of 2.35 [23]. To avoid graphene chemical doping by other materials, the Al_2_O_3_ layer with a relative permittivity $\left(\epsilon_{{\text{Al}}_{2}{\text{O}}_{3}}\right)$ of 2.09 and a thickness of 5 nm is selected as the insulator layers placed above and below the graphene layers [24]. Two semicircular silver nanoribbons are identical with a radius of 100 nm and placed on the top Al_2_O_3_ layer with a gap *g* of 10 nm. The relative permittivity of silver is obtained from Lorentz–Drude model [25, 26]. The graphene layer can be considered as a 2D homogenous anisotropic material and can be modeled as a surface conductivity layer without thickness [17]. The surface conductivity $\left(\sigma_{\text{g}}\right)$ of the single graphene layer is calculated by the Kubo formula [27, 28]:

(1) $$\begin{array}{c} \sigma_{g}\left(\omega ,\mu_{c},\Gamma ,T\right)=\frac{je^{2}}{\pi \hbar^{2}\left(\omega -j2\Gamma \right)}\int_{0}^{\infty }\left(\frac{\partial f_{d}\left(\xi ,\mu_{c},T\right)}{\partial \xi }-\frac{\partial f_{d}\left(-\xi ,\mu_{c},T\right)}{\partial \xi }\right)\xi \text{d}\xi \\ +\frac{je^{2}\left(\omega -j2\Gamma \right)}{\pi \hbar^{2}}\int_{0}^{\infty }\frac{f_{d}\left(\xi ,\mu_{c},T\right)-f_{d}\left(-\xi ,\mu_{c},T\right)}{{\left(\omega -j2\Gamma \right)}^{2}-4\xi /\hbar^{2}}\text{d}\xi \text{,} \end{array}$$

where *ω* is the angular frequency, *μ*_c_ is the Fermi level, *T* is the temperature, the scattering rate Γ = 2*τ*^−1^, the relaxation time *τ* = *μμ*_c_/(*ev*_F_^2^), *μ* is the mobility, *v*_F_ is the Fermi velocity, *e* is the electron charge, *ξ* is the energy, *ћ* is the reduced Planck constant, *k*_B_ is the Boltzmann constant, and the Fermi–Dirac distribution $f_{d}\left(\xi ,\mu_{c},T\right)={\left({\text{e}}^{\left(\xi -\mu_{c}\right)/k_{\text{B}}T}+1\right)}^{-1}$ . In this study, we set *τ* = 0.5 ps and *T* = 300 K [29], [30], [31]. A gate voltage *V*_g_ is applied to the graphene-insulator-graphene capacitor to control the graphene conductivity via electrostatic doping to achieve phase modulation effect, as illustrated in Figure 1a.

Furthermore, the GPM can be fabricated through the typical large-scale graphene synthesis and transfer techniques, and the state-of-art nano processes, such as electron-beam evaporation and nanoimprint lithography [16]. The high quality of the graphene transferring is the key to achieving the high-efficiency phase modulation. The atomic-layer-deposited (ALD) technology can be used for the ultrathin Al_2_O_3_ layer deposition to avoid electric contact between graphene layers and ensure the high electron mobility of graphene [21, 24]. Then the top dual-semicircular-silver-nanoribbon can be etched using laser interference lithography or nanoimprint lithography. Rapid thermal processing is adopted to improve the contact quality between metal electrodes and graphene to reduce the contact resistance, thus improving the modulation bandwidth of the proposed phased modulator.

## 3 Results and discussion

### 3.1 Guiding mode analysis

**Figure 2:**
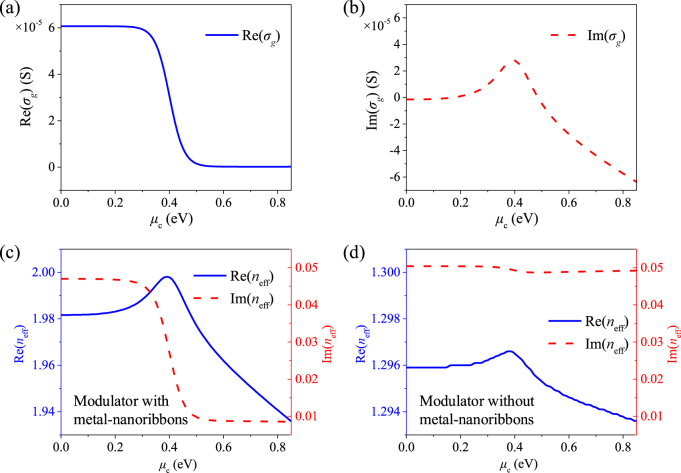
Calculated graphene surface conductivity Re(*σ*_g_) (a) and Im(*σ*_g_) (b) as a function of the graphene chemical potential *μ*_c_. Simulated Re(*n*_eff_) and Im(*n*_eff_) as a function of graphene chemical potential *μ*_c_ for the proposed GPM with metal–nanoribbons (c) and without metal–nanoribbons (d).

**Figure 3:**
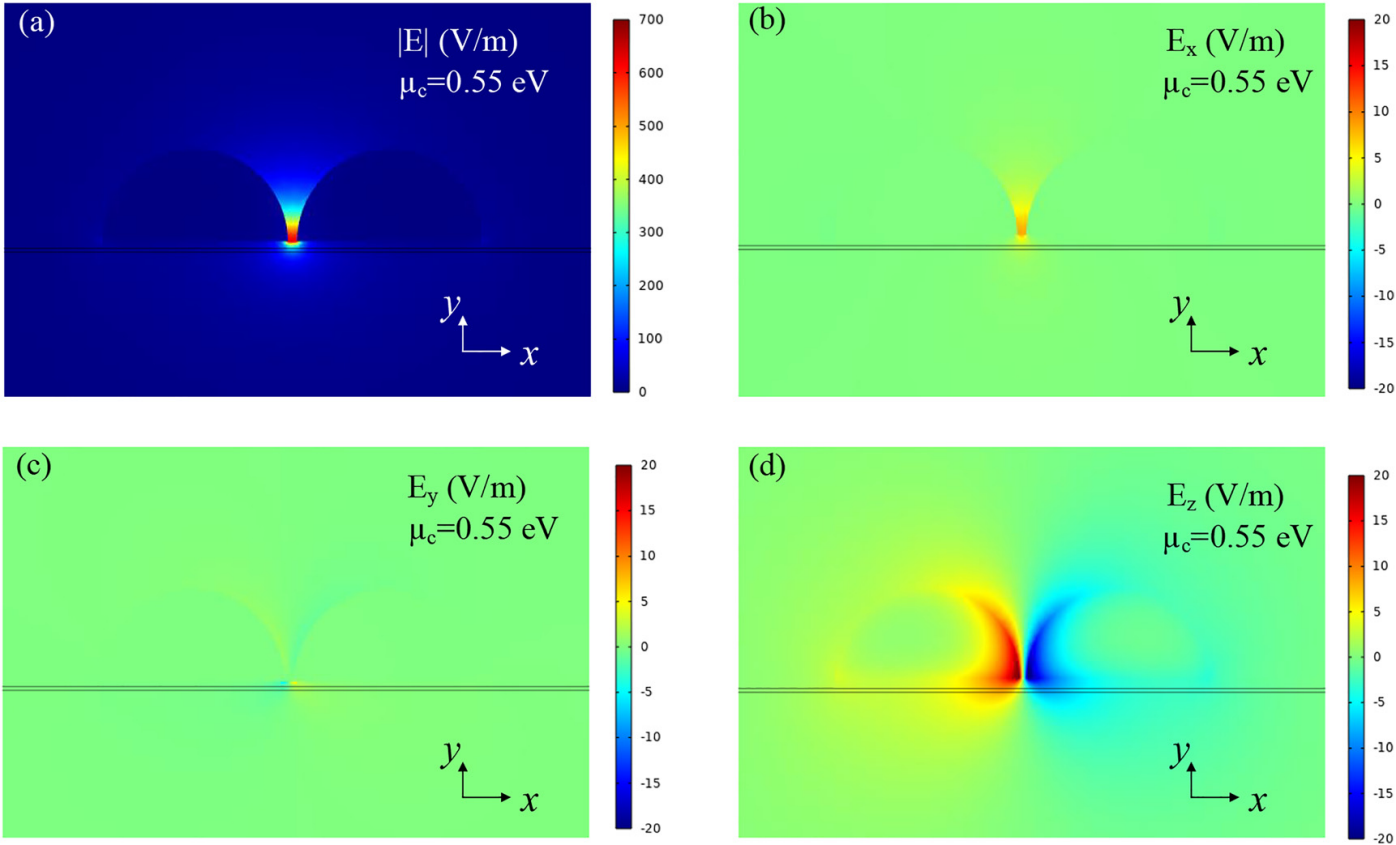
Simulated electric field distributions on the *xy* plane of the GPM with *μ*_c_ = 0.55 eV. (a) Electric field magnitude |E|. (b) Electric field component in *x*-direction (E_*x*_). (c) Electric field component in the *y*-direction (E_*y*_). (d) Electric field component in *z*-direction (E_*z*_).

According to (1), we first analyze the graphene conductivity as a function of graphene chemical potential *μ*_c_ at operating wavelength *λ* = 1.55 μm. As shown in Figure 2a, the Re(*σ*_g_) is very sensitive to *μ*_c_, where Re(*σ*_g_) decreases drastically from 60.7 to 0.17 μS (over 350 times) as *μ*_c_ increases from 0 to 0.85 eV. Figure 2b shows that Im(*σ*_g_) changes from positive to negative by adjusting *μ*_c_ from 0 to 0.85 eV, which implies the properties of graphene switching from “metallic” to “dielectric” states. The great tunability of graphene conductivity indicates the great modulation potential of graphene in the proposed GPMs. To investigate the guiding mode characteristics, we simulate the complex effective refractive index *n*_eff_, attenuation constant *α*, and the field distributions of the proposed GPM on *xoy* plane using the mode analysis solver of COMSOL Multiphysics. The scattering boundary conditions are assigned to all the outer boundaries (enough far away from the center of the modulator) to mimic the free space. The real and imaginary parts of the effective refractive index Re(*n*_eff_) and Im(*n*_eff_) at 1.55 μm under different *μ*_c_ from 0 to 0.85 eV, as shown in Figure 2c. It is found that the modulator can achieve both strong electro-absorptive and electro-refractive effects owning to the huge change range in terms of Re(*n*_eff_) and Im(*n*_eff_). The Im(*n*_eff_) of the modulator drops quickly from 0.047 to 0.009 as *μ*_c_ increases from 0.2 to 0.55 eV. The proposed modulator may operate as an amplitude or intensity modulator when switching *μ*_c_ from 0.2 to 0.55 eV. Especially, it is observed that the Re(*n*_eff_) decreases linearly in a wide range from 1.97 to 1.94 with tiny Im(*n*_eff_) reduces from 0.0089 to 0.0086 as the *μ*_c_ increases from 0.55 to 0.85 eV. The real effective refractive index change ΔRe(*n*_eff_) is 0.03 while the Im(*n*_eff_) change is as small as 0.0003, resulting in a large ΔRe(neff)/ΔIm(neff) of 100 (in the order of 10^2^). That is to say, the light propagation of the modulator undergoes significant phase change with very low optical loss. To investigate the effect of the metal–nanoribbons, the Im(*n*_eff_) of the proposed GPM without metal–nanoribbons are also calculated, as shown in Figure 3d. The ΔRe(*n*_eff_) of the modulator without metal–nanoribbons is about 0.0014 between 0.55 and 0.85 eV, which is much one order smaller than that of modulator with metal–nanoribbons. Meanwhile, the Im(*n*_eff_) remains around 0.049 and the ΔIm(*n*_eff_) is of 0.0004, as well as a small ΔRe(neff)/ΔIm(neff) is of 3.5 between 0.55 and 0.85 eV. Clearly, the modulator with metal–nanoribbons shows much higher ΔRe(neff)/ΔIm(neff) and much smaller Im(*n*_eff_), implying higher phase modulation efficiency and lower insertion loss, compared with the modulator without metal–nanoribbons. Therefore, when switching the *μ*_c_ from 0.55 to 0.85 eV, the proposed modulator with metal–nanoribbons may operate as efficient phase modulator with an excellent phase and amplitude decoupling effect. Here, we only focus on electro-refractive effect-based phase modulation performance in the following discussion.

To better understand the mechanism, the guiding mode field distributions of the GPM at 1.55 μm is analyzed. Figure 3 shows the electric field magnitude |E| and components E_*x*_, E_*y*_, and E_*z*_ distributions (unit: V/m) at 0.55 eV, respectively. Clearly, strong subwavelength confinement is achieved in all cases with intense hybrid SPP fields located around two semicircular silver nano-ribbons gap and closed to the graphene layers. Owing to the integration of dual silver nanoribbons, the hybrid SPP electric fields of GPM is dominantly polarized along the *x*-direction (E_*x*_) and *z*-direction (E_*z*_) but rare polarized in the *y*-direction (E_*y*_), as shown in Figure 3b–d. It is remarkable that the major E_*x*_ and E_*z*_ components are parallel to the graphene plane, allowing the good in-plane mode polarization matching, strong light-graphene interaction, and excellent electro-optic modulation effects.

### 3.2 Phase change and loss characteristics

**Figure 4:**
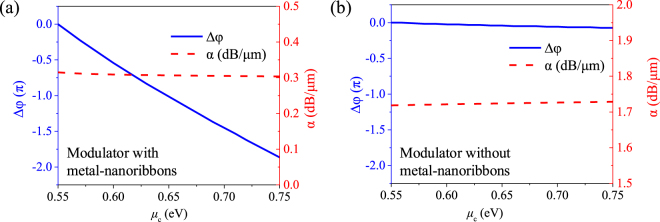
Phase change Δ*φ* and attenuation constant α for the 20 μm length GPM under different chemical potential *μ*_c_ ranging from 0.55 to 0.75 eV at 1.55 μm (a) with the metal–nanoribbons. (b) Without the metal–nanoribbons.

To investigate the modulation characteristics of the proposed GPM, we calculate the phase change (Δ*φ*) and attenuation constant (*α*) as follows:

(2) Δφ=2πλΔRe(neff)L,

(3) α=40πIm(neff)λln10,

where *λ* is the wavelength in free space, and *L* is the length of the modulator. From Figure 2c, the phase change Δ*φ* and the attenuation constant *α* for a 20 μm length GPM without and with metal–nanoribbons as a function of *μ*_c_ at 1.55 μm can be obtained, as shown in Figure 4a and b, respectively. As shown in Figure 4a, when *μ*_c_ is increasing from 0.55 to 0.75 eV, the Δ*φ* of the proposed modulator with metal–nanoribbons decreases almost linearly from 0 to 1.86π with *α* keeps around 0.31 dB/μm. The π phase shift only needs a small *μ*_c_ change of 0.1 eV from 0.55 to 0.65 eV. While, as shown in Figure 4b, the modulator without metal–nanoribbons shows a very small Δ*φ* of 0.073π and a high *α* of ∼1.72 dB/μm, respectively. Clearly, the proposed modulator with the metal–nanoribbons demonstrates an efficient phase modulation with negligible phase and amplitude coupling effect.

**Figure 5:**
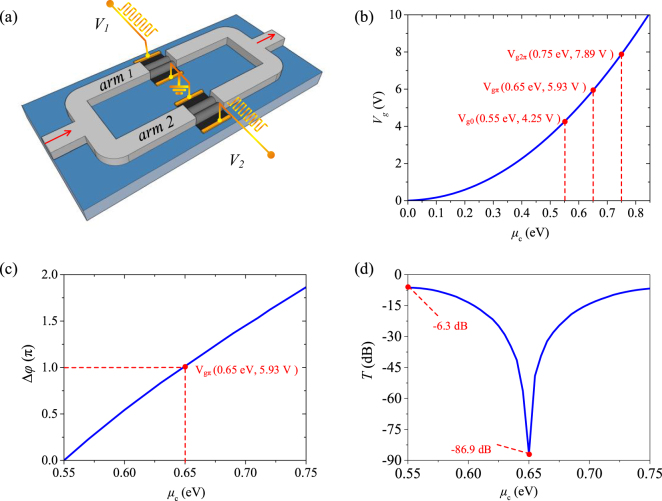
MZ modulator based on GMPs. (a) Schematic of the MZ modulator. (b) The relationship between the gate voltage *V*_g_ and *μ*_c_. (c) Phase difference Δ*φ* as a function of *μ*_c_ at 1.55 μm. (d) Transmission *T* as a function of *μ*_c_ at 1.55 μm.

In an MZ modulator based on the GPM with metal–nanoribbons, as schematically shown in Figure 5a, the optical transmission is given by

(4) $$T\left(\lambda \right)=\frac{1}{4}\left[e^{-\alpha_{1}L}+e^{-\alpha_{2}L}+2e^{-\frac{\alpha_{1}L+\alpha_{2}L}{2}}\;\cos\left(\Delta \varphi \right)\right]$$

where the modulator arm’s length *L* = 20 μm, $\alpha_{1}$ and $\alpha_{2}$ represent the loss of the two arms respectively, and Δ*φ* is the phase difference between two arms when different *μ*_c_ values are applied. The relationship between the gate voltage *V*_g_ and *μ*_c_ is given by $V_{\text{g}}=en_{\text{s}}t/2\epsilon_{0}\epsilon_{{\text{Al}}_{2}{\text{O}}_{3}}$ , where the charge density $n_{\text{s}}=\frac{2}{\pi \hbar v_{F}^{2}}\overset{\infty }{\underset{0}{\int }}\xi \left[f_{\text{d}}\left(\xi ,\mu_{\text{c}},T\right)-f_{\text{d}}\left(\xi ,2\mu_{\text{c}},T\right)\right]\text{d}\xi$ [27]. As shown in Figure 5b, the gate voltage *V*_g_ should increase from 4.25, 5.93, to 7.89 V to obtain the *μ*_c_ ranging from 0.55, 0.65–0.75 eV. By applying *V*_g1_ and *V*_g2_ as 4.25 and 5.93 V for the two modulator arms, the *μ*_c_ difference between them of 0.1 eV is obtained. Figure 5c and d show the phase difference Δ*φ* and transmission *T* of the MZ modulator under different *μ*_c_ at the operating wavelength of 1.55 μm, where the *μ*_c_ one arm is ranging from 0.55 to 0.75 eV while for the other arm is fixed as 0.55 eV. In this case, the extinction ratio (ER) can reach over 80 dB due to the phase difference between two arms approaching π, as shown in Figure 5d. Therefore, the proposed modulator shows high modulation efficiency with V_*π*_L_*π*_ = 5.93 V × 20 μm = 118.67 V μm, which is a significant improvement with respect to previously reported GPMss.

### 3.3 Effect of operating wavelength and geometric parameters

**Figure 6:**
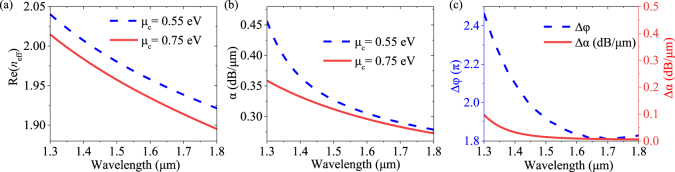
(a)–(c) Dependence of Re(*n*_eff_), *α*, Δ*φ* and Δ*α* of the proposed GPM on the operating wavelength ranging from 1.3 to 1.8 μm.

The effect of optical operating wavelength on the performance of the proposed GPM with metal–nanoribbons is studied. As shown in Figure 6a and b, the modulator’s effective refractive index Re(*n*_eff_) and attenuation constant α under *μ*_c_ = 0.55 and 0.75 eV keep a similar downward trend with wavelength increasing from 1.3 to 1.8 μm. Specially, the Re(*n*_eff_) decreases from 2.04 to 1.92 (from 2.01 to 1.89), and the α decreases from 0.46 dB/μm to 0.28 dB/μm (from 0.36 dB/μm to 0.27 dB/μm) under *μ*_c_ = 0.55 eV (*μ*_c_ = 0.75 eV), respectively. As shown in Figure 6c, the Δ*φ* decreases from 2.46π to 1.82π and the Δ*α* decreases from 0.09 dB/μm to 0.01 dB/μm as the wavelength increases from 1.3 to 1.8 μm. Therefore, the proposed modulator exhibits excellent phase modulation and low insertion loss in an ultra-wide operating wavelength range of 1.3–1.8 μm.

**Figure 7:**
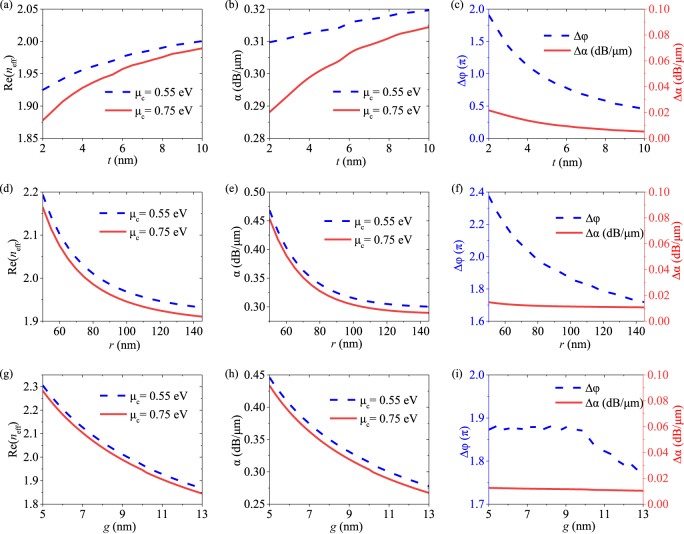
(a)–(c) Dependence of Re(*n*_eff_), *α*, Δ*φ* and Δ*α* on thickness *t* ranging from 2 to 10 nm with *r* = 100 nm and *g* = 10 nm. (d)–(f) Thickness *t* ranging from 2 to 10 nm the radius *r* ranging from 50 to 145 nm with *g* = 10 nm and *t* = 5 nm. (g)–(i) Gap *g* ranging from 5 to 13 nm with *r* = 100 nm and *t* = 5 nm under different *μ*_c_ of 0.55 and 0.75 eV at 1.55 μm, respectively.

The proposed phase modulator is based on hybrid plasmonic waveguides with dual semicircular silver nano-ribbons and graphene- ${\text{Al}}_{2}{\text{O}}_{3}$ -graphene capacitor. Here, we further investigate the effects of the key geometrical parameters such as insulator spacer thickness (*t*), the semicircle radius (*r*), and the gap (*g*) on the modulator’s effective refractive index Re(*n*_eff_), attenuation *α*, the 20 μm-length modulator phase change Δ*φ*, and Δ*α* under *μ*_c_ of 0.55 and 0.75 eV at the operating wavelength of 1.55 μm, as shown in Figure 7. As is shown in Figure 7a–c, the Re(*n*_eff_) and *α* increase, Δ*φ* (Δ*α*) decreases from 1.9π to 0.45π (from 0.022 dB/μm to 0.005 dB/μm) as *t* increases from 2 to 10 nm. The interaction between the propagating light and graphene will be weaker as *t* increases, resulting in a decline in phase change and modulation efficiency. In Figure 7d–i, the Re(*n*_eff_), *α* and Δ*φ* keep a similar downward trend as *r* and *g* increase. The Δ*φ* remains large phase change over 1.7π while the Δ*α* remains negligible level from 0.015 dB/μm to 0.01 dB/μm between 0.55 and 0.75 eV in the whole *r* and *g* ranges, implying the proposed phase modulator has good phase modulation without obvious amplitude modulation. In this work, the parameters *t*, *r* and *g* for the modulator are selected as 5, 100, 10 nm for a moderate trade-off between the modulation efficiency, insertion loss, and fabrication complexity.

### 3.4 Modulation bandwidth

**Figure 8:**
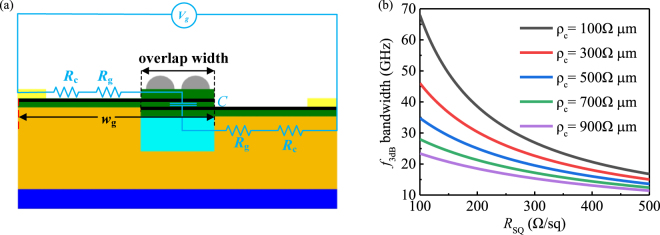
(a) The equivalent circuit of the proposed GPM. (b) $f_{3dB}$ bandwidth as a function of *R*_SQ_ and *ρ*_c_, where the geometry and dielectric combination are the same as those in Figure 1.

Bandwidth is one of the key parameters for the optical phase modulator, which can be used to evaluate the modulation speed. To further study broadband properties, we calculate the bandwidth (*f*_3dB_) with an equivalent circuit model as shown in Figure 8a. The modulation process can be equivalent to the switching states between the charge and discharge of the graphene-insulator-graphene capacitor. The modulator’s total *RC*-limited bandwidth is given by

(5) $$f_{3dB}=\frac{1}{2\pi \left(2R_{\text{c}}+2R_{\text{g}}\right)C}\text{,}$$

where *R*_g_ is graphene resistance, *R*_c_ is contact resistance, and C is the capacity of the graphene-insulator-graphene capacitor. The *R*_g_ is usually defined through *R*_SQ_ in the unit of resistance per square, which is related to carrier mobility of graphene and typically located between 100 and 500 Ω/sq [32, 33]. The high *R*_c_ is determined by the contact resistivity *ρ*_c_, which was shown in the range of 100–1000 Ω μm experimentally [34], [35], [36]. And the capacity value is related to permittivity $\epsilon_{{\text{Al}}_{2}{\text{O}}_{3}}$ , capacitor area *S* and distance *t*. Their values can be calculated by

(6) $$R_{\text{g}}=R_{\text{SQ}}\frac{w_{\text{g}}}{L}\text{,}$$

(7) $$R_{\text{c}}=\frac{\rho_{\text{c}}}{L}\text{,}$$

(8) $$C=\frac{\epsilon_{0}\epsilon_{{\text{Al}}_{2}{\text{O}}_{3}}s}{t}\text{,}$$

where *w*_g_ (3.2 μm) is the width of graphene, *L* (20 μm) is the length of the modulator, $\epsilon_{0}$ is the permittivity, the overlap width of graphene layers is 0.6 μm, *S* (12 μm^2^) is the area of the capacity, and *t* (5 nm) is the distance between two graphene sheets. Therefore, *C* is 55.8 fF, and the bandwidth can be calculated from Eq. (5), as shown in Figure 8b. The 3 dB modulation bandwidth can be up to 67.96 GHz when *ρ*_c_ is 100 Ω μm. Notably, *L* does not impact the 3 dB modulation bandwidth but has a significant influence on phase change and insertion loss. Furthermore, in terms of energy consumption, the energy per bit is calculated by: *E*_b_ = *C*Δ*V*^2^/4, where the difference value of voltage to achieve phase modulation Δ*V* = *V*_g*π*_ − *V*_g0_ = 1.68 V. In this case, the phase modulator shows a low energy consumption of about 157.49 fJ/bit and a small footprint of 12 μm^2^, which is promising for near-infrared modulation applications.

**Figure 9:**
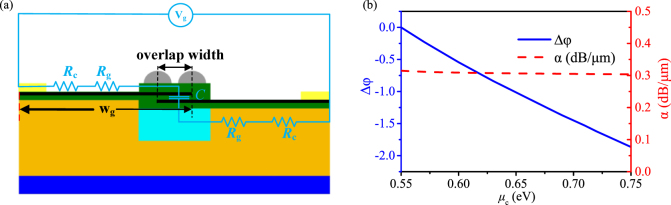
Improved GPM with reduced overlap width of the graphene capacitor. (a) Equivalent circuit of the improved modulator. (b) Δ*φ* and *α* as functions of *μ*_c_ of the improved modulator.

The modulation bandwidth can be further improved by reducing the graphene overlap width of the graphene–Al_2_O_3_–graphene capacitor to reduce the capacity. The improved phase modulator with reduced overlap width is shown in Figure 9a. When the overlap width of graphene is reduced from 600 to 110 nm, the bandwidth will increase from 67.9 to 370.36 GHz under *R*_SQ_ = 100 Ω/sq and *ρ*_c_ = 100 Ω μm. It is remarkable that the electric field of the modulator is mainly confined in the small metal-ribbon gap area above the graphene layers, ensuring the strong interaction between the field and graphene layers. Therefore, the reduction of overlap width will greatly reduce the *C* and increase the bandwidth without affecting the phase modulation performance. As shown Figure 9b, this improved phase modulator shows Δ*φ* of 1.82π and *α* of 0.3 dB/μm with *L* = 20 μm as the *μ*_c_ switches from 0.55 to 0.75 eV, which is very close to the original design with Δ*φ* of 1.86π and *α* of 0.3 dB/μm as shown in Figure 4. Clearly, this improved phase modulator may be applied to the integrated circuits for wider bandwidth or faster modulation speed.

### 3.5 Comparison of different phase modulators

To better demonstrate the advantage of the proposed design, we present the performance comparison of the proposed phase modulator with some recently reported works, as shown in Table 1. For example, Mohin et al. experimentally demonstrated a GPM with a large V_*π*_L_*π*_ of 3 × 10^5^ V μm, which results from the weak light and graphene interaction [21]. Sorianello et al. proposed an electro-refractive modulator based on single or double-layer graphene on top of silicon waveguides experimentally and numerically showing a V_*π*_L_*π*_ of 2800 V μm and a bandwidth of 5 GHz [5], as well as a V_*π*_L_*π*_ of 1600 V μm and a bandwidth of 30 GHz [13]. Shu et al. experimentally proposed a graphene-based silicon MZ modulator with V_*π*_L_*π*_ of 1290 V μm [19]. To achieve small V_π_, Mao et al. proposed an integrated phase modulator based on graphene-Si photonic crystal waveguide with a small V_*π*_L_*π*_ of 2150 V μm but need a huge modulation L_π_ of 2870 μm [20].

**Table 1:** A comparison of some recent reported GPMs.

	References	Length (μm)	V_*π*_L_*π*_ (V μm)	3 dB modulation bandwidth (GHz)	Energy consumption (fJ/bit)	Operating wavelength (μm)
Experimental results	Mohin et al. [21]	200	3 × 10^5^	–	–	1.53–1.57
	Sorianello et al. [5]	300	2800	5	–	1.55
	Shu et al. [19]	40	1290	–	–	1.55
Numerical results	Sorianello et al. [13]	500	1600	30	0.38	1.55
	Mao et al. [20]	2870	2150	67	–	1.55
	Yuki et al. [22]	227	450	–	–	4
	This work: initial structure	20	118.67	67.96	157.49	1.3–1.8
	Improved structure	20	120.50	370.36	30.22	1.3–1.8

Here, the proposed phase modulator shows excellent performance with the length (L_π_) of 20 μm, high modulation efficiency with the V_*π*_L_*π*_ of 118.67 V μm, large 3 dB modulation bandwidth of 67.96 GHz, small energy consumption of 157.49 fJ/bit, and wide optical operating wavelength range of 1.3–1.8 μm. By reducing the overlap width of the graphene-Al_2_O_3_-graphene capacitor, the improved modulator demonstrates an ultra-wide modulation bandwidth of 370.36 GHz and extremely low energy consumption of 30.22 fJ/bit. Therefore, these proposed graphene modulators possess obvious advantages compared with the recent designs, which may have extensive potential applications in low energy consumption integrated silicon-based platforms.

## 4 Conclusions

In this work, we proposed a new variety of ultra-compact high-efficiency GPMs based on hybrid plasmonic waveguides for near-infrared applications. We numerically study the key characteristics of the proposed modulators including the guiding mode, phase change, transmission loss, modulation efficiency, bandwidth, and energy consumption, as well as the effect of operating wavelength and geometric parameters. It is found that owing to the mode polarization matching and strong light-graphene interaction, the proposed GPM possesses excellent performance with a high modulation efficiency with V_*π*_L_*π*_ of 118.67 V μm and a low energy consumption of 157.49 fJ/bit, which is superior to the recently reported graphene-based modulators. By reducing the overlap width of the graphene–Al_2_O_3_–graphene capacitor, the modulation bandwidth and energy consumption of the modulator can be further improved to 370.36 GHz and 30.22 fJ/bit, respectively. These modulators also show a good phase and amplitude decoupling effect. This study may offer a new way to design efficient electro-absorptive or electro-refractive modulators, which may have potential applications in various high-speed telecommunications, interconnects, and other graphene-based integrated photonic devices.
